# Analysis of viral diversity in stool samples from infants and children with acute gastroenteritis in Kuwait using Metagenomics approach

**DOI:** 10.1186/s12985-020-1287-5

**Published:** 2020-01-30

**Authors:** Hawraa Adel Mohammad, Nada Mohammed Madi, Widad Al-Nakib

**Affiliations:** Virology Unit, Department of Microbiology, Faculty of Medicine, Kuwait University, P.O.Box 24923, 13110 Safat, Kuwait

**Keywords:** Next-generation sequencing, Metagenomics approach, Viral gastroenteritis, Kuwait

## Abstract

**Background:**

Current molecular target-dependent methods are used to detect only known viruses. However, metagenomics based on next-generation sequencing (NGS) technique is a target-independent assay that enables simultaneous detection and genomic characterisation of all microorganisms present in a sample. In this study, we aimed to develop a metagenomics approach using NGS to identify and characterise viruses in stool samples from infants and children with Acute Gastroenteritis (AGE) in Kuwait.

**Methods:**

We have investigated 84 stool samples from infants and children aged one month to ten years old with signs and symptoms of gastroenteritis who attended Mubarak Al-Kabeer and Al-Amiri hospitals in Kuwait from January to December 2017. A metagenomics approach using NGS to characterise viruses in clinical samples was used. Also, the commercial Real-Time PCR assay was used to detect viruses causing gastroenteritis.

**Results:**

Metagenomics analysis revealed an average of 280,768 reads in which 5% of the reads were derived from viruses. The analysis of viral sequences verified that single infection of human adenovirus was the leading cause of gastroenteritis among infants and children, which was detected in 23.2% of the patients, followed by a mixed infection of human adenovirus and other viruses, which was detected in 20.9% of patients. Also, the newly discovered viruses known to cause gastroenteritis were detected, such as astrovirus MLB2, primate bocaparvovirus-1, Aichivirus A, cardiovirus, parechovirus A, astrovirus VA4, cosavirus-F, and bufavirus-3. Our results showed 71% agreement (k = 0.445, *P* = 0.000) between multiplex Real-Time PCR, which is used as a routine diagnostic test and metagenomics approach in the detection of viruses causing gastroenteritis in clinical samples.

**Conclusion:**

Despite the difficulties in sample preparation and analysis process, we showed that metagenomics approach is a powerful and promising tool for the detection and characterisation of different viruses in clinical samples.

## Background

The traditional methods for virus discovery such as filtration, tissue culture, electron microscopy and serology were powerful techniques for the detection of viruses. However, due to their limitations, the traditional techniques were replaced by molecular techniques such as polymerase chain reaction (PCR) and DNA sequencing (Sanger method) [1]. Although these target-dependant molecular techniques had the credits in the discovery of many viruses, many new and novel human viruses are not yet revealed [2]. Therefore, innovative approaches that overcome the limitations of conventional methods for the detection of viruses in clinical samples are needed [3]. In 1998, the word metagenome was first used to define the collection of uncultivable microorganisms in a soil sample [4]. Now, this term is used to describe the characteristics of the recovered information from genomes directly from a sample [5–7]. Metagenomics approach based on NGS has been used to detect different infectious agents in different samples [8]. The first application of metagenomics study in virus discovery was the analysis of virus particles in soil samples from marine sites in San Diego [9]. Now, the metagenomics approach is used widely in different research areas, including marine ecological research, plant and agriculture, human genetics and diagnosis of human diseases [10]. Virologists were the first to use the metagenomics approach to detect viruses, causing different diseases in humans such as respiratory tract infections, acute flaccid paralysis in children, and gastroenteritis [8, 11, 12].

Gastroenteritis is the second leading cause of death among infants and children worldwide [13]. It is responsible for approximately three million deaths each year, causing high morbidity and mortality rate globally [14, 15]. Although at least 25 different bacteria and protozoa can cause diarrhoea, more than 75% of cases found to be caused by viruses [16]. Viruses that can cause gastroenteritis include rotavirus, norovirus, enteric adenovirus, human astrovirus, and Sapporo virus [17, 18]. Currently, the diagnostic methods used to detect viruses in stool samples are sequence-dependent molecular amplification techniques such as PCR, which cannot identify a pool of viruses and completely new viruses in clinical samples. Therefore, a novel approach that is sequence-independent such as viral metagenomics approach using NGS is desirable and should be developed for viral diagnosis and to overcome the unresolved cases of gastroenteritis.

## Materials and methods

Eighty-four infants and children with signs and symptoms of gastroenteritis, including diarrhoea, vomiting, fever, and abdominal pain from January to December 2017 were enrolled in this study. The patients aged between 1 and 10 years old (median age = 2 years old) who attended Al-Amiri and Mubarak Al-Kabeer Hospitals in Kuwait. Fresh stool samples were collected from the patients and stored in − 80 °C for further processing. Patient’s demographics were retrieved from the laboratory requests. Also, ten fresh stool samples were collected from healthy children aged between 1 and 5 years old (median age = 3.5 years old). The collected samples from patients and healthy children were processed at the Virology Unit and Research Core Facility/OMICS Research Unit, Health Sciences Centre, Kuwait University, for the presence of viruses causing gastroenteritis by metagenomics approach using NGS and the commercial multiplex Real-Time PCR assay.

### Nucleic acids extraction

The collected stool samples were re-suspended in phosphate buffer saline, incubated for 1 h at 4 °C and centrifuged at 6000 Xg (5530 rpm) for 5 min at 4 °C to enrich the viruses. Total nucleic acids were extracted from stool samples using automated MagNA Pure LC system (Roche Diagnostics, Indianapolis, USA), according to the manufacturer’s instructions.

### Next-generation sequencing and metagenomics analysis

The extracted nucleic acids (RNA and DNA) were processed for metagenomics analysis using the Illumina MiSeq (San Diego, CA, USA) platform for NGS according to standard procedures [15]. Briefly, the genomic host DNA in the extracted nucleic acids was removed using Ambion DNA-free (Invitrogen, ThermoFisher Scientific, USA)) following the manufacturer’s instructions. QuantiTect® Whole transcriptome, (Qiagen, Valencia, CA, USA) was used for the synthesis of single-strand cDNA from the DNA free RNA primed by random hexamers and then amplified according to the manufacturer’s instructions. Quantification of cDNA was performed using Qubit^R^ Fluorometer and Qubit™ dsDNA BR Assay Kit (Invitrogen, California, USA) following the manufacturer’s instructions and one ng of cDNA was used for library preparation. DNA libraries were prepared using Illumina TruSeq DNA Library Preparation Kit V2 (Illumina San Diego, CA, USA). The pooled DNA libraries were sequenced using the Illumina MiSeq instrument at Research Core Facility/OMICS Research Unit, Health Science Centre, Kuwait University, to generate 150-bp paired-end reads.

### Bioinformatics

After sequencing by Miseq sequencer, fastq sequence files were checked for quality using Fastqc (Andrews, 2014) and low quality ends, below 20 and above 240, were trimmed using FASTX-Toolkit (http://hannonlab.cshl.edu/fastx_toolkit/). The genome sequence of human, viruses, and bacteria was downloaded from NCBI (National Center for Biotechnology Information) RefSeq, and a custom database was created using build option in Kraken software. Each paired-end fastq file was mapped to the database using Kraken (Wood and Salzberg, 2014) to assign taxonomic labels to the sequences. For a detailed analysis of the reads, fastq files were also mapped to the database using BWA-MEM (Burrows-Wheeler Alignment mem option) (Li and Durbin, 2009) and sam files were obtained for the alignment and were filtered for MAPQ score 0. Samtools flagstat was used for the information about the percentage of reads aligned to each database (Li et al., 2009a). The sam files generated from the BWA program was analyzed by bbmap (https://jgi.doe.gov/data-and-tools/bbtools/) for detailed analysis. Raw reads data with high-quality reads were archived at NCBI (https://www.ncbi.nlm.nih.gov/) as sequence read archive (SRA) with BioProject accession number: PRJNA587350.

### Multiplex real-time PCR assay

New nucleic acid extractions from each sample were prepared and used for multiplex Real-Time PCR. Fast Track Diagnostic Kit (Fast-Track Diagnostic, Luxemburg, Germany) was used to detect viruses causing AGE according to the manufacturer’s instructions. The assay is a routine diagnostic test which is performed at the Virology Unit, Mubarak Al-Kabeer Hospital, Kuwait, for the detection of viruses that cause gastroenteritis including norovirus genotype 1 and 2; astrovirus; rotavirus; adenovirus; and sapovirus. All runs were performed using the LightCycler®480 instrument II (Roche Diagnostic, Mannheim, Germany).

### Statistical analysis

The data were analysed using computer software “Statistical Package for Social Sciences”, SPSS version 25.0 (IBM Corp, Armonk, NY). The descriptive statistics were presented as frequencies and percentages. Cohen’s Kappa statistics (*k*) was applied to find the agreement by both metagenomics and multiplex Real-Time PCR assays. Two-tailed probability value *P < 0.05* was considered statistically significant.

## Results

### Characteristics of the study population

Between January–December 2017, a total of 84 stool samples from infants and children were collected from two hospitals in Kuwait; Al-Amiri and Mubarak Al-Kabeer Hospitals. Among these patients, 59 (70.2%) were males, and 25 (29.7%) were females, 44 (52.4%) of the patients were non-Kuwaiti, and 40 (47.6%) were Kuwaiti. The median age of the patients was two years old. At the time of admission, the patients were complaining of different clinical presentations of gastroenteritis; 67.8% (*n* = 57) of the patients had diarrhoea, 13.0% (*n* = 11) had bloody diarrhoea, 3.5% (*n* = 3) had fever, 1.1% (*n* = 1) had vomiting, and 14.2% (*n* = 12) had other symptoms such as abdomen pain, sepsis, and weight loss. As a control measure, ten fresh stool samples from healthy children were included in this study, 3 (30%) of the children were females, and 7 (70%) were males. The children aged 1–5 years old (median age = 3.5 years).

### Virus detection by metagenomics approach

**Fig. 1:**
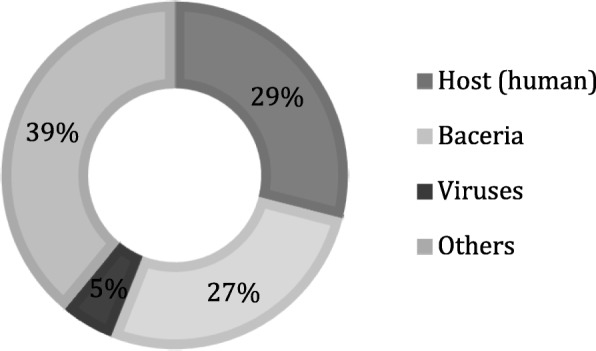
Doughnut chart of the average values of metagenomics sequencing reads in the clinical samples

From the total of 94 stool samples collected from patients with gastroenteritis and healthy individuals, cDNA was obtained to perform whole-genome sequencing using Illumina Miseq sequencer for the detection of RNA and DNA viruses causing gastroenteritis. Sequencing of cDNA libraries pooled from all samples generated an average of 280,768 reads (range; 404–1,001,170) after quality filtering and trimming. On an average; 29% (range; 0.04–88.35%) of the reads were originated from host (human) genome; 27% (range; 2.25–70.03%) of the reads belonged to bacteria genome, 5% (range; 0.02–25.02%) of reads derived from viruses; and the remaining reads (39%) were obtained from other organisms that could not be matched to any of the known in the database (Fig. 1).

**Fig. 2:**
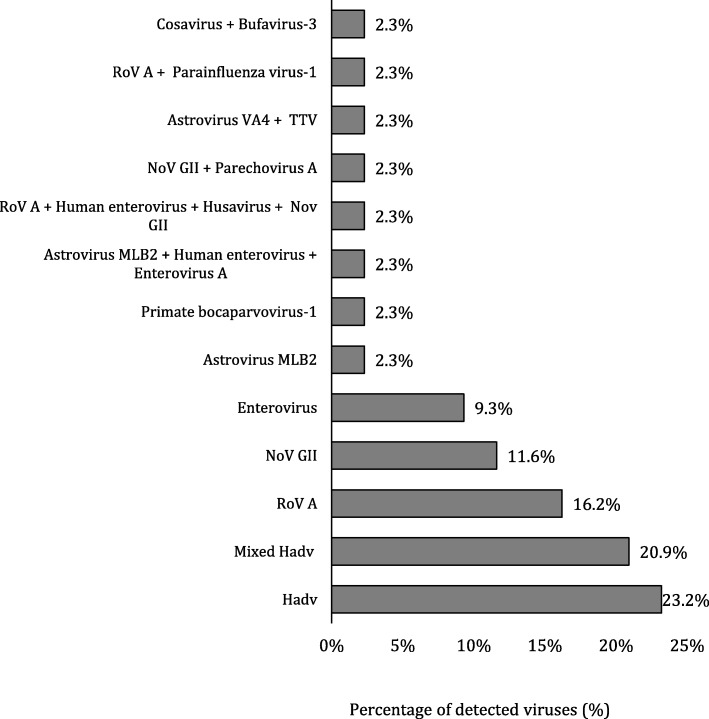
Clustered Bar chart of the percentage of patients positive for viruses causing gastroenteritis and non-gastroenteritis viruses detected by metagenomics sequencing approach (*n* = 84). RoV, Rotavirus; NoV GII, Norovirus GII; Hadv, Human adenovirus; TTV, Torque teno virus

Viral sequences identified from metagenomic sequencing approach after alignment using Kraken software were originated from human, animal and plant-associated viruses. Human viruses causing gastroenteritis were found in 43 out of 84 (51.1%) patients tested, and they were as follow (Fig. 2): human adenovirus (10; 23.2%), mixed infection of human adenovirus and other viruses (9; 20.9%), rotavirus A (7; 16.2%), norovirus GII (5; 11.6%), enteroviruses (4; 9.3%). Moreover, metagenomics sequencing approach exhibited its feasibility to detect the newly discovered viruses causing gastroenteritis despite the low number of reads obtained, and these viruses were as follow: astrovirus MLB2 was found in one (2.3%) patient; primate bocaparvovirus-1 was found in one (2.3%) patient; mixed infection of primate bocavirus-1 and adenovirus was found in one (2.3%) patient; mixed infection of astrovirus MLB2, human enterovirus, and enterovirus A was found in one (2.3%) patient. Furthermore, other new viruses were found as a mixed infection with other viruses, and each combination was detected in one (2.3%) patient (Fig. 2, Table 1): Aichi virus; cardiovirus (saffold); parechovirus A; astrovirus VA4; cosavirus F; and bufavirus-3. In addition to viruses causing gastroenteritis, non-gastroenteritis viruses were detected by metagenomics analysis, and they presented as mixed infection; husavirus was detected in one (2.3%) patients, torque teno virus was detected in one (2.3%) patients, and parainfluenza virus-1 was detected in one (2.3%) patient. The metagenomics analysis revealed the presence of different genotypes of viruses such as different types of human adenovirus (A-G), enterovirus (A-D), and norovirus GII in the samples (Table 1). Along with patients with AGE, stool samples from ten healthy children were tested; three were positive for gastroenteritis viruses; one child had human adenovirus C, the second had rotavirus A, and the third had astrovirus MLB2 (Table 1).

**Table 1:** Study population characteristics and results of Multiplex Real-time PCR and Metagenomics approach (*n* = 94)

Sample No.	Age	Gender	Metagenomics sequencing results	No. of Reads	Multiplex Real-Time PCR results	CT value
1	1 yr	F	Rotavirus A	1	Rotavirus	26.48
2	2 yr	F	–		–	
3	3 yr	M	–		–	
4	2 mo	M	–		–	
5	2 mo	M	–		–	
6	6 yr	M	Human adenovirus E	4	–	
7	2 yr	F	–		–	
8	2 yr	M	Astrovirus MLB2	4	Human adenovirus	25.73
9	1 mo	M	–		–	
10	2 yr	M	–		–	
11	2 yr	M	Human adenovirus E	1	Human adenovirus	31.99
12	1 yr	M	–		Rotavirus	14.53
					Human adenovirus	28.68
13	3 yr	M	–		–	
14	3 yr	M	–		–	
15	7 mo	M	–		Norovirus GII	16.33
16	3 yr	M	–		–	
17	1 yr	M	–		Rotavirus	18.61
					Human adenovirus	30.20
					Sapporo virus	33.30
					Norovirus GII	28.57
18	1 yr	M	Human adenovirus C	12	Human adenovirus	23.96
			Human adenovirus E	2	Norovirus GII	17.33
			Norovirus GII	11		
19	4 yr	F	Enterovirus C	587	–	
			Enterovirus B	452		
			Enterovirus D	1		
20	3 yr	M	–		Rotavirus	25.90
					Human adenovirus	20.05
21	2 yr	F	Norovirus GII	2	Rotavirus	31.62
			Human adenovirus C	1	Human adenovirus	33.00
22	1 yr	M	Primate bocaparvovirus-1	1	Rotavirus	32.65
23	1 yr	M	–		–	
24	4 yr	M	–		–	
25	1 yr	M	Human adenovirus C	1	Rotavirus	26.36
26	2 mo	F	–		–	
27	3 mo	M	Human enterovirus	2	–	
28	3 mo	M	–		–	
29	1 yr	M	Enterovirus B	3	–	
Sample No.	Age	Gender	Metagenomics sequencing results	No. of Reads	Multiplex Real-Time PCR results	CT value
30	4 mo	M	Human adenovirus F	744	Human adenovirus	12.31
			Human adenovirus C	47		
			Human adenovirus B	9		
31	3 yr	F	–		–	
32	4 yr	M	Human adenovirus F	550	Human adenovirus	10.62
			Human adenovirus B	12		
			Human adenovirus E	4		
			Human adenovirus C	4		
			Human adenovirus D	4		
33	2 yr	M	Rotavirus A	41	Rotavirus	16.40
			Human adenovirus C		Human adenovirus	26.55
				1		
34	2 yr	M	Astrovirus MLB2	1272	Human adenovirus	32.47
			Human enterovirus	1	Norovirus GII	24.99
			Enterovirus A	1		
35	4 yr	F	–		Rotavirus	30.78
					Human adenovirus	27.83
36	2 yr	M	Human adenovirus B	1	Human adenovirus	21.38
37	5 yr	F	Human adenovirus F	2	–	
38	4 yr	M	–		–	
39	1 yr	M	Rotavirus A	5	Rotavirus	14.65
			Human enterovirus	1	Human adenovirus	24.82
			Husavirus	2		
			Norovirus GII	1		
40	8 mo	M	Human adenovirus F	436	Human adenovirus	12.48
			Human adenovirus C	9		
41	4 yr	M	Human adenovirus B	1	Rotavirus	32.64
			Husavirus	1	Human adenovirus	19.34
42	4 yr	M	–		–	
43	7 mo	F	–		Rotavirus	14.83
					Human adenovirus	32.30
44	2 yr	M	Rotavirus A	3	Rotavirus	16.99
			Human adenovirus B	1	Human adenovirus	32..82
45	2 yr	F	Cardiovirus (saffold)	8	Human astrovirus	32.51
			Human adenovirus E	1	Human adenovirus	32.88
			Torque teno virus-3	4		
			Torque teno virus-19	1		
46	4 yr	M	–		Human adenovirus	26.44
47	2 yr	F	–		Rotavirus	19.81
					Human adenovirus	28.67
Sample No.	Age	Gender	Metagenomics sequencing results	No. of Reads	Multiplex Real-Time PCR results	CT value
48	2 yr	F	Rotavirus A	105	Rotavirus	22.31
49	3 yr	F	–		Rotavirus	29.60
					Human adenovirus	29.52
50	1 mo	M	–		–	
51	4 mo	M	–		–	
52	2 yr	F	Rotavirus A	2	Rotavirus	16.72
					Human adenovirus	29.55
53	1 yr	M	–		–	
54	1 yr	M	–		Rotavirus	32.55
55	5 yr	M	Rotavirus A	2	Rotavirus	12.85
					Human adenovirus	32.62
56	1 yr	F	–		Rotavirus	21.22
					Human adenovirus	32.30
57	10 mo	M	Rotavirus A	40	Rotavirus	17.84
58	2 mo	F	Human adenovirus A	1	Rotavirus	32.22
59	1 yr	M	Norovirus GII	10	Norovirus GII	15.87
			Parechovirus A	1	Sapporo virus	18.42
					Human adenovirus	32.51
60	1 yr	M	Rotavirus A	4	Rotavirus	16.78
			Human adenovirus B	1	Human adenovirus	26.70
			Aichi virus A	1	Norovirus GII	27.57
61	3 yr	M	Primate bocaparvovirus 1	6	Rotavirus	16.34
			Human adenovirus B	1	Human adenovirus	29.83
			Human adenovirus G	1	Norovirus GII	28.49
			Norovirus GII	1		
62	8 mo	M	–		Human adenovirus	32.87
					Norovirus GII	31.84
63	3 yr	M	–	–	Rotavirus	20.27
					Human adenovirus	30.71
					Norovirus GII	28.94
64	1 yr	M	Rotavirus A	13	Rotavirus	18.31
65	3 yr	F	Astrovirus VA4	2	Human adenovirus	32.63
			Torque teno virus	1	Norovirus GI	30.00
66	2 yr	M	Rotavirus A	5	Rotavirus	20.58
			Parainfluenza virus 1	10		
67	4 yr	M	–		–	
68	7 yr	F	Human adenovirus B	5	–	
69	1 mo	F	–	–	–	
70	3 mo	M	–	–	–	
71	3 yr	M	–	–	–	
72	6 yr	M	Cosavirus F	1	Human adenovirus	31.84
			Bufavirus-3	1		
73	4 yr	F	Norovirus GII	1	–	
Sample No.	Age	Gender	Metagenomics sequencing results	No. of Reads	Multiplex Real-Time PCR results	CT value
74	1 yr	M	Rotavirus A	18	Rotavirus	14.76
					Human adenovirus	29.54
					Sapporo virus	27.25
75	3 mo	M	–	–	–	
76	1 mo	M	–		Human adenovirus	33.00
77	4 mo	M	Enterovirus C	11	–	
78	1 yr	F	–	–	Human astrovirus	31.73
79	5 mo	F	Norovirus GII	3	Norovirus GII	16.96
80	6 mo	M	Norovirus GII	621	Norovirus GII	13.57
					Human adenovirus	32.77
81	2 yr	F	Human adenovirus C	159	Rotavirus	30.97
			Norovirus GII	26	Human adenovirus	23.34
					Norovirus GII	16.51
82	2 yr	F	Norovirus GII	5	Norovirus GII	19.25
83	2 yr	M	Norovirus GII	33	Norovirus GII	16.88
84	5 yr	M	–	–	Rotavirus	19.36
85	2 yr	F	–	–	–	
86	1 yr	M	–	–	–	
87	3 yr	F	–	–	–	
88	5 yr	M	Husavirus	1	–	
89	4 yr	M	–	–	–	
90	2 yr	M	–	–	–	
91	4 yr	M	Human adenovirus C	1	Rotavirus	16.52
					Human adenovirus	31.35
92	4 yr	F	–	–	–	
93	4 yr	M	Rotavirus A	3	Rotavirus	16.44
94	2 yr	M	Astrovirus MLB2	1	–	

The number of viral reads obtained from metagenomics approach raged from 1 to 1272, and the highest number of reads was for Astrovirus MLB2. The high-quality reads were archived at NCBI as SRA with accession numbers presented in the Additional file 1.

### Virus detection by multiplex real-time PCR

**Fig. 3:**
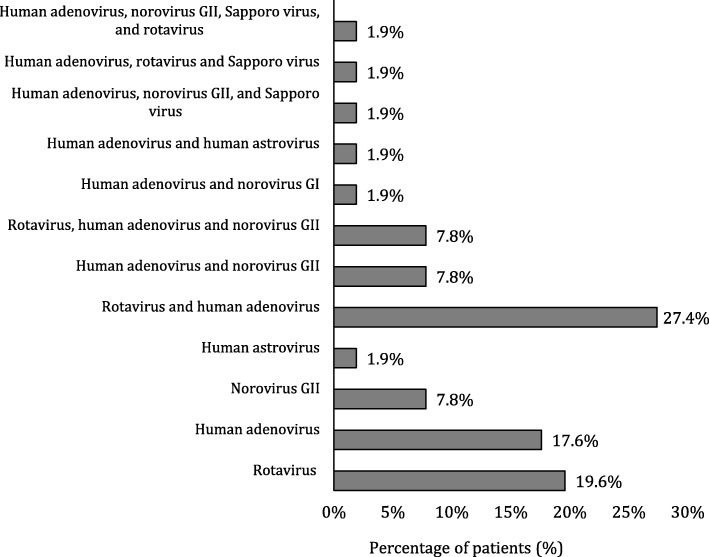
Clustered Bar chart of the percentage of patients positive for viruses causing gastroenteritis detected by multiplex RT-PCR assay (*n* = 51)

The 84 stool samples from patients with symptoms of gastroenteritis were also tested for the presence of viruses by multiplex Real-Time PCR assay. The results demonstrated that 51 (60.7%) of the patients were positive for viruses causing gastroenteritis and 33 (39.2%) of the patients were negative. Amongst the positive patients, 14 patients (27.4%) had mixed infection of rotavirus, and human adenovirus, ten patients (19.6%) had rotavirus, nine patients (17.6%) had human adenovirus, four patients (7.8%) had a norovirus GII, and one patient (1.9%) had human astrovirus. Also, other patients had mixed viral infection, but in low percentage (Fig. 3). On the other hand, out of the ten healthy children tested, two were positive for gastroenteritis viruses; one child had rotavirus, and the other one had a mixed infection of rotavirus and human adenovirus. The threshold cycle value (C_T_) for each detected virus is shown in Table 1.

### Comparison between multiplex real-time PCR and metagenomics approach

To evaluate the capability of the metagenomics approach to detect viruses causing gastroenteritis, the results of multiplex Real-Time PCR and metagenomics sequencing approaches were compared (Table 1). Of the 94 stool samples, 53 (56%) samples were positive for viruses causing gastroenteritis by Real-Time multiplex PCR and 41 (44%) samples were negative. However, using metagenomic sequencing approach, 46 (49%) samples were positive for viruses causing gastroenteritis, and 48 (51%) samples were negative. Generally, 30 (32%) samples were detected as positive for the same viruses, and 37 (39.3%) as negative by both methods, resulting in an absolute agreement on 67 (71%) samples. Regarding the negative samples, it is important to mention that the positive metagenomics samples other than those detected by Real-Time multiplex PCR were considered as negative. By applying the Cohen’s Kappa statistics for a measure of agreement, the result gave moderate agreement between the two methods (k = 0.445, *P* = 0.000).

## Discussion

In this study, we demonstrated the potential of metagenomics approach using high-throughput NGS method to reveal viruses present in stool samples from AGE cases in infants and children in Kuwait between January– December 2017. This study is the first of its kind in Kuwait to perform metagenomics analysis using NGS to detect various viruses causing gastroenteritis in stool samples from infants and children with signs and symptoms of gastroenteritis. Stool samples from 94 patients with gastroenteritis and healthy children were first analysed for the presence of viruses by metagenomic approach using NGS. The results showed that cDNA libraries generated an average of 280,768 reads of 150-bp paired-end reads. Among the total reads, 29% of the reads were originated from the host (human), 27% of the reads were obtained from the bacterial genome, while only 5% of reads were derived from viruses. In comparison to our results, Yang and colleagues identified a higher average (91.6%) of host (human) genome reads, and 61.5% were from the bacterial genome in the clinical samples [19]. However, Madi and colleagues identified an average of 64% of the host (human) genome reads in respiratory samples [20]. In our study, the viral reads were higher in comparison to other studies; Yang and colleagues identified only 3.1% of viral reads [19], while Madi and coworkers identified a lower average of viral reads (2%) [20]. On the other hand, other studies of viral metagenomics in stool samples have recorded better viral reads; 76% [21], 35.6% [22], and 23% [23]. Probably, the two-steps of DNase treatment of the samples which was performed in this study have resulted in the enrichment of the viral particles and reduced the host genome.

After the assembly of the contigs generated by NGS, the results showed that 43 out of 85 (51%) stool samples collected from infants and children with AGE were positive for viruses known to cause gastroenteritis. Human adenovirus was the predominant virus detected in stool samples (23.2%), and the combined infection of human adenovirus and other viruses was the second most prevalent infection (20.9%). However, rotavirus A was the third predominant virus detected in stool samples (16.2%) followed by norovirus GII (11.6%). By a study in southern Brazil to investigate viruses causing gastroenteritis in hospitalised pediatric patients, the results showed that out of 225 fecal samples tested, human adenovirus was the predominant virus (16%), while 8% of the samples were positive for norovirus, and 6% were positive for rotavirus [24]. Additionally, many studies have shown that human enteric adenovirus types 40 and 41 (HAdV-40, AHdV-41) are an important cause of gastroenteritis [25–28]. In contrary to our study, updated estimates on diarrhoea-related childhood revealed that rotavirus is the leading cause of diarrhoea among children worldwide [29–32]. A previous study in Kuwait has investigated the prevalence of viruses causing gastroenteritis in stool samples from children aged up to five years with acute gastroenteritis using an electron microscope and enzyme immunoassay. The results showed that rotavirus was the primary (40%) virus causing diarrhoea among children in Kuwait [33, 34]. Concerning norovirus, other studies have also demonstrated that norovirus GII is the predominant genogroup known to cause gastroenteritis in children [35–38].

One of the features of the metagenomic approach is its ability to determine different genogroups and subtypes of viruses. In this study, different genotypes of adenovirus (A-G) and enterovirus (A-D) were detected. Furthermore, the approach detected norovirus GII as the main genogroup presented in the samples.

The results of the metagenomic analysis in this study have demonstrated that mix viral infection is a common phenomenon in patients with gastroenteritis (Fig. 2). In our study, 15 out of 43 (35%) stool samples from patients with gastroenteritis had mixed infection with one or more enteric viruses. Adenoviruses were detected as co-infection viruses in 20.9% (9/43) of the positive samples. According to several studies conducted in Korea, Japan, Albania, and Venezuela, mixed infections of enteric and non-enteric adenoviruses are common and might play a role in acute gastroenteritis [39–43]. The clinical importance of mixed viral infection is an unresolved question. However, mixed viral infections have biological and epidemiological implications. Viruses in mixed infections may interact synergistically or antagonistically altering the concentration of either or both viruses and accordingly affecting the outcome of the disease. We speculate that some viruses which are causing gastroenteritis to depend on other viruses for their pathogenicity. However, this concept requires further investigations.

The most interesting clinical finding for this study was the detection of many newly discovered viruses, which might be associated with gastroenteritis, and they were as follow; primate bocaparvovirus-1, astrovirus MLB2, astrovirus VA4, cardiovirus (saffold), parechovirus A, Aichi virus A, cosavirus F, and bufavirus-3. It is noteworthy that this study is the first study in Kuwait to detect these novel viruses in stool samples using a metagenomics approach.

Gastroenteritis samples were analysed further using multiplex Real-Time PCR using FTD kit as a comparison and reference test for the presence of viruses causing gastroenteritis in stool samples from patients and healthy children. In our study, the results showed that 60.7% of patients were positive for viruses causing gastroenteritis disease. Of the positive patients, 53% had mixed viral infections. Mixed infection of adenovirus and rotavirus was the predominant (27.4%). A similar percentage of mixed infection of human adenovirus and rotavirus (27.2%) was reported in a study conducted in Albania to assess the occurrence of human adenovirus in children with acute gastroenteritis symptoms [42]. In our study, rotavirus was the most predominant virus detected by multiplex Real-Time PCR as a single infection (19.6%). However, this percentage is lower than the percentage of rotavirus recorded previously in Kuwait [33, 34]. We speculate that the reduced incidence of rotavirus among children is due to the implementation of rotavirus vaccine in Kuwait.

The comparison of the metagenomic sequencing data and that of multiplex RT-PCR revealed an absolute moderate agreement of 71% (k = 0.445). The divergence between the results obtained by multiplex Real-Time PCR and metagenomics approach could be explained by the fact that multiplex Real-Time PCR is a standardised technique that is routinely used for the detection of the commonly known viruses associated with gastroenteritis. On the contrary, the metagenomics approach is a newly introduced technology in Kuwait and requires further standardisations and validations in order to use it as a routine diagnostic test. The question is how to intercept metagenomics analysis results by NGS in term of what is clinically relevant for patients requires further investigations. Although metagenomics did not detect all multiplex Real-Time PCR positive samples, it did provide several advantages over the multiplex Real-Time PCR assay. For instance, the metagenomics approach detected several known and newly discovered viruses among children with gastroenteritis for the first time in Kuwait that could not be identified by the multiplex Real-Time PCR method. Moreover, the metagenomic approach had the advantage to detect different genogroup of viruses, for example, adenovirus, enterovirus and norovirus genotypes. Also, unlike multiplex Real-Time PCR, the metagenomic approach can detect completely new viruses.

This study had many limitations — first, the small samples size of both patients with AGE and healthy children. Second, low total reads and viral reads obtained by NGS, which can be improved by enrichment of virus content in the samples.

## Conclusion

In conclusion, the metagenomic-sequencing approach was successfully implemented to detect viruses in infants and children with gastroenteritis in Kuwait. We showed that metagenomics analysis holds promise as a diagnostics tool, where multiplex Real-Time PCR could not identify many viruses known to cause gastroenteritis but were detected by metagenomics approach. Although metagenomics has provided a powerful tool for detecting the newly discovered viruses that cause gastroenteritis, the detection of these viruses is not sufficient to prove causality. Based on these findings, we cannot rule out these viruses as the cause of acute gastroenteritis in infants and children. Therefore, further studies in the immediate future with a larger sample size of healthy controls and causes of AGE are needed to evaluate their clinical significance.

To become a routine diagnostic tool, the approach requires further improvement in sample preparation, validation of pipelines for reads sorting and taxonomic assignation, lower the prices of the machine and reagents, and standardisation. These developments will expedite the feasibility of metagenomics approach and allow its implementation in every diagnostic laboratory.

## Supplementary information

**Additional file 1.** Accession numbers of the highest quality reads at NCBI as SRA

## Data Availability

All relevant data are within the paper.

## Abbreviations

AGE: Acute gastroenteritis
FTD: Fast track diagnostic
NGS: Next-generation sequencing
PCR: Polymerase chain reaction
