# Supplementary material for: Analysis of viral diversity in stool samples from infants and children with acute gastroenteritis in Kuwait using Metagenomics approach
Source: Virol J. 2020 Jan 30;17:10. doi: 10.1186/s12985-020-1287-5 (PMC6993487; doi:10.1186/s12985-020-1287-5)
Supplement: Supplementary file 1 — Additional file 1. Accession numbers of the highest quality reads at NCBI as SRA [file 12985_2020_1287_MOESM1_ESM.docx]

Additional file 1 Accession numbers of the highest quality reads at NCBI as SRA.

| **SAMPLE NUMBER** | **ACCESION NO.** |
| --- | --- |
| 32 | SRX7090119 |
| 60 | SRX7090096 |
| 59 | SRX7090095 |
| 45 | SRX7090094 |
| 34 | SRX7090093 |
| 33 | SRX7090092 |
| 30 | SRX7090090 |
| 19 | SRX7090089 |
| 80 | SRX7090088 |
| 72 | SRX7090087 |
| 65 | SRX7090086 |
| 61 | SRX7090085 |
| 18 | SRX7090084 |
| 6 | SRX7090083 |
